# Initial Mix-and-Match COVID-19 Vaccination Perceptions, Concerns, and Side Effects across Canadians

**DOI:** 10.3390/vaccines10010093

**Published:** 2022-01-09

**Authors:** Adam Palanica, Jouhyun Jeon

**Affiliations:** Klick Applied Sciences, Klick Health, Toronto, ON M4W 3R8, Canada; cjeon@klick.com

**Keywords:** COVID-19, vaccine, perceptions, vaccine concerns, vaccine side effects

## Abstract

Research indicates that mixing the first two doses of COVID-19 vaccine types (i.e., adenoviral vector and mRNA) produces potent immune responses against the coronavirus, but it is unclear how individuals may perceive these benefits, or whether there are different concerns compared to individuals who received two doses of the same vaccine. This research examines the demographic characteristics, psychological perceptions, and vaccination-related opinions and experiences of a large Canadian sample (*N* = 1002) who had received two initial doses of any COVID-19 vaccine combination. Participants included 791 (78.9%) who received two doses of the exact same brand and type of vaccine, 164 (16.4%) who received two doses of the same type of vaccine (i.e., either mRNA or adenoviral vector) but from different brands (e.g., Pfizer-BioNTech + Moderna), and 47 (4.7%) who received two doses from different types and brands of vaccine (e.g., Oxford-AstraZeneca + Pfizer-BioNTech). Results showed that, after the first vaccine dose, participants who received an adenoviral vector vaccine (e.g., Oxford-AstraZeneca) experienced the highest number of common side effects, and more severe levels of each side effect compared to those who received an mRNA vaccine (e.g., Pfizer-BioNTech or Moderna). After the second dose, participants who received Moderna as their second vaccine experienced the highest number of and most severe side effects, regardless of whether they received Moderna, Pfizer-BioNTech, or Oxford-AstraZeneca as their first dose. Real-world implications of these findings are discussed.

## 1. Introduction

Research indicates that mixing and matching the first two COVID-19 vaccine doses (i.e., receiving heterologous vaccine types) produces an increased immune response against the coronavirus compared to receiving two doses of the same (homologous) vaccine [1,2,3,4,5]. For example, studies have shown that vaccinating people with a first dose of Oxford-AstraZeneca (ChAdOx1; adenoviral vector) followed by Pfizer-BioNTech (BNT162b2; mRNA) produces a potent immune response against COVID-19 [6]. Additionally, due to the risk of discontinuation of specific vaccines, struggles in the production and transportation of vaccines, and difficulties supplying enough vaccine doses in low-income countries or regions, mix-and-match vaccination has been considered as a means to overcome the COVID-19 pandemic [7].

Mixing COVID-19 vaccines can be a beneficial way to entice individuals with added protection when faced with safety concerns and unpredictable supplies of a specific vaccine they received in the past [8]. Because of safety concerns with the Oxford-AstraZeneca vaccine [9,10,11], several countries have recommended that individuals who received a first dose of this vaccine should get a different vaccine for their second dose [6]. However, it is unclear how mixing and matching vaccines produces sustained levels of efficacy and mitigates against potential side effects in the population. Furthermore, the perceptions of individuals are not well studied when it comes to how they may perceive the benefits or concerns of mixing and matching vaccines compared to those who receive two doses of the same vaccine. Although the attitudes, acceptance, and hesitancy toward COVID-19 vaccines have been well studied recently [12,13,14], it is still unclear how the perceptions and experiences of individuals differ after they have received either the same two vaccine doses, or two different types of vaccines.

Since Canada was one of the few countries to implement a mix-and-match COVID-19 vaccine dosing structure across the population, this study examined a large Canadian sample (*N* = 1002) intended to be representative of the different vaccine participants. This research utilized an online survey to examine the opinions and perceptions of participants who received two initial doses of any COVID-19 vaccine combination. The purpose was to investigate any differences in perceived benefits, concerns, or self-reported side effects from participants who received two initial doses of the same COVID-19 vaccine versus those who received a mix-and-match vaccine combination, and whether there were any differences across demographics of age, gender, ethnicity, education, religion, lifestyle, or health status. This research has important implications for general audiences, healthcare providers, scientists, government and health officials, and those who are interested in understanding participant perceptions during the COVID-19 pandemic, and in applying this knowledge to future disease outbreaks.

## 2. Methods

### 2.1. Participants

This study included 1002 Canadian participants from the online survey platform Prolific (https://prolific.co/ (accessed on 21 December 2021)). This sample size yields approximately a 3.1% margin of error with a 95% confidence interval for the entire Canadian population (~38 million), or alternatively, a 4.1% margin of error with a 99% confidence interval for the Canadian population.

The only inclusion criteria included adults, aged 18+ years, and those who received two initial COVID-19 vaccine doses. The study also implemented a 50/50 split between males and females to gather an equal distribution of responses across genders. Participants in this study resided in 548 different geographical regions (i.e., based on different first 3 postal code digits) across all 10 provinces in every major region of Canada.

Of the total sample, 791 (78.9%) participants received two doses of the exact same brand and type of vaccine, 164 (16.4%) received two doses of the same type of vaccine (i.e., either mRNA or adenoviral vector) but from different brands (e.g., (Pfizer-BioNTech + Moderna) or (Oxford-AstraZeneca + Janssen [Johnson & Johnson])), and 47 (4.7%) received two doses from different types and brands of vaccine (e.g., Oxford-AstraZeneca + Pfizer-BioNTech). These three study groups (i.e., Same Brand, Different Brand, Different Type) were the main independent variables of the investigation (Table 1).

### 2.2. Survey Design

The online survey was designed with reference to recent studies on attitudes and opinions toward COVID-19 vaccines [12,13,14], and overall face and content validity was developed in consultation with medical scientists and healthcare experts in the field. The survey was organized to include various demographic questions (socioeconomic factors), medical information (BMI, health issues, perceived overall health status), COVID-19 history (awareness of the disease, infection experience), and personal experiences regarding the 1st and 2nd dose of the COVID-19 vaccine (type of vaccine received, concerns before each dose, side effects after each dose, perceived efficacy and safety of the vaccine treatment). All questions were closed-ended and listed in multiple-choice format, with several options as responses. For example, the list of concerns regarding vaccine doses included:

- *Do not like needles;*
- *Allergic to COVID-19 vaccine;*
- *Potential side effects after COVID-19 vaccination;*
- *Overall safety of vaccines in general;*
- *Lack of scientific research or evidence on COVID-19 vaccine effectiveness;*
- *Concerns about how quickly the COVID-19 vaccine was developed and tested;*
- *Do not know enough about the COVID-19 vaccine;*
- *Concerns about negative long-term effects of COVID-19 vaccination;*
- *Concerns about getting COVID-19 from the vaccine;*
- *Concerns that the COVID-19 vaccine will cause permanent changes to my DNA;*
- *Concerns that the COVID-19 vaccine is not effective;*
- *Concerns that the COVID-19 vaccine will not work against new variants or mutations;*
- *Belief that if others get the COVID-19 vaccine, then I will not need to;*
- *Belief that the COVID-19 outbreak is as not as serious as people say it is;*
- *Belief that my own natural immune system is safe against COVID-19.*

Potential side effects after each vaccine dose were listed with a Likert scale of 0 = “*did not experience*”, 1 = “*mild*”, 2 = “*severe*”, 3 = “*severe and hospitalized*”, and included:

- *Pain, redness, or swelling on the arm where you got the needle;*
- *Muscle or body pain (e.g., joint pain, muscle aches, or soreness);*
- *Abdominal pain;*
- *Chest pain or pressure;*
- *Tiredness or fatigue;*
- *Fever;*
- *Headache;*
- *Chills;*
- *Nausea;*
- *Shortness of breath;*
- *Cough;*
- *Sore throat;*
- *Loss of taste or smell.*

Participants were also asked about their perceived safety against contracting COVID-19 (i.e., the likelihood of being immune to the disease) after receiving each vaccine dose, on a scale of 1 = “*extremely high*”, 2 = “*high*”, 3 = “*moderate*”, 4 = “*low*”, 5 = “*none*”.

Each main question also contained an “*other*” response option at the bottom of the answers if participants chose to respond with an open-ended option that was not listed. However, preliminary analyses revealed no unusual responses from participants, so these were coded as part of the closed-ended responses.

### 2.3. Procedure

Participants were gathered from a cross-sectional, nationally representative sample of Canada using the online survey platform Prolific (https://prolific.co/ (accessed on 21 December 2021)). The survey was circulated in November 2021. Each participant was paid $0.80 USD to complete a ~5–6 min survey assessing their opinions and perceptions of their COVID-19 vaccine experiences. All participants signed informed written consent and the study received full ethics clearance from Canadian SHIELD Ethics Review Board (https://cserb.com/ (accessed on 21 December 2021)).

### 2.4. Data Analysis

Since this study was exploratory in nature, the main purpose was to examine any differences in demographics, perceptions, concerns, and self-reported side effects of receiving two initial doses of the same COVID-19 vaccine type versus those who received a mix-and-match vaccine combination. Therefore, independent and paired-samples *t*-tests (two-tailed) were used to analyze differences between and within the three different study groups: Same Brand (e.g., Pfizer-BioNTech + Pfizer-BioNTech), Different Brand (e.g., Pfizer-BioNTech + Moderna), and Different Type (e.g., Oxford-AstraZeneca + Pfizer-BioNTech). That is, head-to-head comparisons were analyzed between: (i) Same Brand vs. Different Brand, (ii) Same Brand vs. Different Type, and (iii) Different Brand vs. Different Type. Pearson correlations were also used to analyze vaccine concerns and side effects across participant age. A *p*-value of <0.05 was considered statistically significant for all results. Data were analyzed using SPSS version 23.

## 3. Results

### 3.1. Demographics

No significant differences were found between Same Brand and Different Brand participants in terms of gender, age, ethnicity, marital status, having children, amount of people in household, education status, employment status, household income, religious status, body mass index (BMI), perceived overall health, having any major health issues, likelihood of receiving an annual influenza vaccine, perceived knowledge and information regarding COVID-19, having been infected with COVID previously, concerns before getting either vaccine dose, or perceptions of safety after either vaccine dose (all *p* > 0.12).

Different Type individuals were significantly different from Same Brand and Different Brand individuals in terms of being older, more likely to be married and have children, more likely to follow some type of religion, have a higher household income, and also more likely to receive an annual influenza vaccine (all *p* < 0.05). There was also a significant difference of ethnicity, as the majority of Different Type participants were White/Caucasian (39 of 47; 83%) compared to other backgrounds. These factors seemed to be a confound with overall age, since all of these variables were significantly correlated with each other. No other significant demographic differences were found across study groups.

### 3.2. Concerns before Receiving 1st Vaccine

Before the first vaccine dose, 49% of participants across groups reported no concerns at all, with no differences between groups (all *p* > 0.28). Of the 51% of participants that did have concerns, no significant differences were found in the total amount of concerns between Same Brand (*M* = 1.49), Different Brand (*M* = 1.24), or Different Type (*M* = 1.06) individuals (all *p* > 0.10), with the most common concerns being either “*potential side effects after COVID-19 vaccination*” (32.8%), “*do not like needles*” (18.0%), “*concerns about negative long-term effects of COVID-19 vaccination*” (18.0%), “*concerns about how quickly the COVID-19 vaccine was developed and tested*” (15.3%), or “*concerns that the COVID-19 vaccine will not work against new variants or mutations*” (14.9%).

### 3.3. Concerns before Receiving 2nd Vaccine

Before the second vaccine dose, 57% of participants across groups reported no concerns at all, with no differences between groups (all *p* > 0.29). Of the 43% of participants that did have concerns, no significant differences were found in the total amount of concerns between Same Brand (*M* = 1.03), Different Brand (*M* = 0.92), or Different Type (*M* = 0.89) participants (all *p* > 0.33), with the most common concerns being either “*potential side effects after COVID-19 vaccination*” (28.6%), “*do not like needles*” (14.6%), “*concerns about negative long-term effects of COVID-19 vaccination*” (11.9%), or “*concerns that the COVID-19 vaccine will not work against new variants or mutations*” (9.8%).

Paired-samples *t*-tests within each group revealed that Same Brand and Different Brand participants had significantly fewer concerns before their second vaccine dose (Same Brand *M* = 1.03; Different Brand *M* = 0.92) compared to before their first vaccine dose (Same Brand *M* = 1.49; Different Brand *M* = 1.24; all *p* < 0.05), yet Different Type participants had equal levels of concern before receiving their first (*M* = 1.06) and second doses (*M* = 0.89; *p* > 0.38).

### 3.4. Self-Perceived Safety after Vaccination

Individuals from all three groups felt significantly safer against contracting COVID-19 after their second vaccine dose (*M* = 2.17) than after their first vaccine dose (*M* = 2.87), from a scale of 1 = “*extremely high*” to 5 = “*none*” (all *p* < 0.0001). No significant differences between groups were found with regard to perceived safety after either vaccine dose.

### 3.5. Self-Reported Side Effects after 1st Vaccine

After the first vaccine dose, only 11% of participants across groups reported no side effects at all, with the majority (89%) reporting at least one side effect.

No significant differences in total side effects were experienced between Same Brand (*M* = 2.62 symptoms) and Different Brand (*M* = 2.48 symptoms) participants, with individuals from both groups reporting some level of pain in the arm where they got the needle (73.6%), tiredness/fatigue (56.1%), muscle/body pain (50.2%), or headache (27.1%). No significant differences were found in the severity level of these symptoms between Same Brand and Different Brand groups, with most participants feeling mild symptoms.

Different Type participants experienced significantly more total side effects (*M* = 4.28 symptoms) than Same Brand and Different Brand participants (all *p* < 0.001), with significantly more severe levels of symptoms of tiredness/fatigue, muscle/body pain, headache, chills, fever, nausea, and abdominal pain (all *p* < 0.05; see Table 2).

In terms of serious adverse effects of vaccination that required hospitalization, participants responded to each potential side effect experience using a Likert scale of 0 = “*did not experience*”, 1 = “*mild*”, 2 = “*severe*”, 3 = “*severe and hospitalized*”. In total, two participants (0.2%) responded with “3” (i.e., severe and hospitalized) after the 1st vaccination; one participant for a sore throat and one for a headache. Both of these individuals received Pfizer-BioNTech for their 1st vaccine.

### 3.6. Self-Reported Side Effects after 2nd Vaccine

After the second vaccine dose, only 13% of participants across groups reported no side effects at all, with the majority (87%) reporting at least one side effect.

No significant differences in total side effects were experienced between Same Brand (*M* = 3.14 symptoms) and Different Type (*M* = 3.32 symptoms) participants, with participants from both groups reporting some level of pain in the arm where they got the needle (70.9%), tiredness/fatigue (64.3%), muscle/body pain (56.7%), headache (40.1%), or fever (30.4%).

Different Brand participants (*M* = 4.04 symptoms) experienced significantly more total side effects than Same Brand participants, and more severe levels of symptoms, including tiredness/fatigue, muscle/body pain, headache, chills, and fever (all *p* < 0.05). Different Brand participants also experienced marginally more total side effects than Different Type participants (*p* = 0.099) and more severe levels of symptoms of chills and fever (all *p* < 0.05; see Table 3).

Paired-samples *t*-tests (to examine within-participant differences) also revealed that participants from all three groups experienced significantly more total side effects after their second vaccine dose (Same Brand *M* = 3.14; Different Brand *M* = 4.04; Different Type *M* = 3.32) compared to after their first vaccine dose (Same Brand *M* = 2.62; Different Brand *M* = 2.48; Different Type *M* = 4.28; all *p* < 0.05).

In terms of serious adverse effects of vaccination that required hospitalization, five (0.5%) participants responded with “3” (i.e., severe and hospitalized) after the 2nd vaccination, for pain in the arm where they got the needle, tiredness/fatigue, muscle/body pain, headache, fever, and/or nausea. All of these participants were Same Brand participants, with three who received a Pfizer-BioNTech + Pfizer-BioNTech combination, and two who received a Moderna + Moderna combination.

### 3.7. Differences in Side Effects between Vaccine Brands

After the first vaccine, Different Type participants experienced the most amount of total side effects (*M* = 4.28), yet after the second vaccine, Different Brand participants experienced the most amount of side effects (*M* = 4.04). To examine this finding further, *t*-tests were performed between vaccine brands within each study group to identify any differences in reported side effects.

After the first vaccine, Same Brand participants who received Moderna reported significantly more total side effects (*M* = 3.17) than those who received Pfizer-BioNTech (*M* = 2.43; *p* < 0.001; Table 4), with significantly more severe levels of symptoms of pain in the arm where they got the needle, muscle/body pain, headache, chills, fever, and nausea (all *p* < 0.05). No significant differences were found between Same Brand participants who received Oxford-AstraZeneca (*M* = 2.94) versus Moderna or Pfizer-BioNTech.

Different Brand participants reported no significant difference in first vaccine total side effects between Moderna (*M* = 2.44) and Pfizer-BioNTech (*M* = 2.49). There was not enough statistical power to examine any other differences in vaccine brands within groups (Table 1 and Table 4).

After the second vaccine, Same Brand participants who received Moderna reported significantly more total side effects (*M* = 4.58) than those who received Pfizer-BioNTech (*M* = 2.70; *p* < 0.001), with significantly more severe levels of symptoms of pain in the arm where they got the needle, tiredness/fatigue, muscle/body pain, headache, chills, fever, nausea, sore throat, and cough (all *p* < 0.05; Table 4). Same Brand participants who received Moderna also reported significantly more total side effects than those who received Oxford-AstraZeneca (*M* = 2.31; *p* < 0.001; Table 4), with significantly more severe levels of symptoms of pain in the arm where they got the needle, tiredness/fatigue, muscle/body pain, and headache (all *p* < 0.05).

Different Brand participants who received Moderna reported significantly more second vaccine total side effects (*M* = 4.33) than those who received Pfizer-BioNTech (*M* = 2.70; *p* < 0.005), with significantly more severe levels of symptoms of pain in the arm where they got the needle, headache, chills, and fever (all *p* < 0.05).

No significant difference was found for Different Type participants who received Moderna or Pfizer-BioNTech (*p* > 0.19), but this may be due to a lack of statistical power, since visual inspection of Table 4 shows a higher number of total side effects for Moderna.

### 3.8. Differences in Concerns and Side Effects between Age and Gender

Across the entire sample, age was significantly, but weakly, negatively correlated with the total amount of reported side effects after the 1st dose (Pearson’s correlation *r* = −0.07, *p* < 0.05) and 2nd dose (Pearson’s correlation *r* = −0.14, *p* < 0.001). This indicates that younger individuals tended to report more total side effects than older individuals.

Additionally, small but significant differences in gender were also found. Female participants expressed more total concerns (*M* = 1.61) than male participants (*M* = 1.24) before the 1st dose (independent *t*-test, *p* < 0.01). Females also reported more total side effects than males after the 1st dose (*M* = 2.88 vs. 2.46) and after the 2nd dose (*M* = 3.63 vs. 2.96; all *p* < 0.005).

## 4. Discussion

### 4.1. Main Findings

This study examined the perceptions and experiences of a large representative Canadian sample of participants who received any initial two-dose combination of the government-approved COVID-19 vaccines (i.e., Pfizer-BioNTech, Moderna, and Oxford-AstraZeneca). Overall, 79% of participants received two of the same brand and type of vaccine, which was mostly comprised of Pfizer-BioNTech and Moderna mRNA vaccines. Another 16% of participants received two of the same type of vaccine coming from different brands, which again was mostly a Pfizer-BioNTech (1st) + Moderna (2nd) mRNA combination. Lastly, 5% of participants received a completely different combination of brand and type of vaccine, which was mostly comprised of an Oxford-AstraZeneca adenoviral vector + either Pfizer-BioNTech or Moderna mRNA vaccine. According to the latest results from the Government of Canada [15], these results are fairly representative of the rest of the population.

No major differences in demographic factors were found across groups, other than the fact that participants who received the adenoviral vector + mRNA vaccine combination tended to be older and more likely to be married, have children, and have a higher income. This finding seems reasonable since older individuals, who were the initial eligible population of the COVID-19 vaccine, were likely to have received Oxford-AstraZeneca as one of the first approved vaccines in Canada, and before any potential side effects (e.g., blood clots with low platelets) were identified. Health Canada was originally unaware that Oxford-AstraZeneca would cause potential thrombotic complications, so when these were findings were reported, the Canadian population started to adopt mRNA vaccines. Thus, those who received the adenoviral vector vaccine first were more likely to switch to an mRNA dose later on when Pfizer-BioNTech and Moderna became more readily available, especially due to the safety concerns and risks of Oxford-AstraZeneca in younger adults (under 55) in Canada and other countries across the world [16,17,18].

Most participants did not have many concerns before receiving either vaccine dose, with an average of ~1 to 1.5 concerns, most commonly oriented around potential side effects. Participants also felt a moderate to high level of safety with regard to efficacy against contracting COVID-19, and this increased with two vaccine doses as opposed to one.

After each vaccination, nearly 90% of participants reported some side effects, with an average of ~2.5 to 4 symptoms for each participant. The most common side effects included pain in the arm where participants got the needle, tiredness/fatigue, muscle/body pain, headache, chills, and/or fever. After the first dose, participants who received an adenoviral vector vaccine (e.g., Oxford-AstraZeneca) experienced the highest number of total side effects, and more severe levels of the abovementioned symptoms. For the second dose, very few people received an adenoviral vector vaccine, and most participants from all study groups received an mRNA vaccine. Results suggest that participants who received Moderna as their second vaccine experienced the highest number and most severe levels of side effects, regardless of whether they received Moderna, Pfizer-BioNTech, or AstraZeneca as their first dose. Taken together, these findings suggest that both brand and type of COVID-19 vaccine have influential effects on reported side effects and experiences of participants. Adenoviral vector vaccines may induce more side effects than mRNA types at first, but as a second booster shot, different brands of mRNA vaccine can also affect negative side effects.

With regard to serious adverse effects of vaccination, a handful of participants reported that they required hospitalization after the 1st and 2nd vaccinations, either for pain in the arm where they got the needle, tiredness/fatigue, muscle/body pain, headache, fever, sore throat, and/or nausea. All these individuals were Same Brand participants, who received either Pfizer-BioNTech or Moderna after their 1st and/or 2nd vaccination. Although this only represents less than 1% of the sample, future research should examine serious side effects of vaccination in the greater population.

### 4.2. Limitations

One limitation of this study is the self-report nature of the results. Participants could be unconsciously or implicitly biased in their self-narratives, and may be influenced by news or social media stories of the pandemic. For example, loss of taste or smell, cough, or sore throat may be an unlikely side effect of a COVID-19 vaccine, and may in fact indicate that participants got infected (unknowingly) with COVID-19 within a short timeframe after their vaccination. It is unclear whether this was the case, whether these rare vaccine side effects actually mimicked symptoms from a COVID-19 infection, or whether these self-reported observations represent a “nocebo effect”. Nevertheless, it is assumed that these anonymous participants would have no benefit to lie about their vaccination history, and the large sample size of the study should have mitigated any potential outlier responses. As a second limitation, this study only analyzed participants in Canada due to the convenience of gathering mix-and-match COVID-19 vaccine individuals. However, this research could be replicated in other countries to examine whether similar trends exist. Lastly, with the arrival of a third vaccine booster shot [19], future research could investigate the perceptions, opinions, and experiences of individuals who receive completely different vaccines across all three doses.

### 4.3. Conclusions

The findings of this research have important implications for scientists and public health officials in other countries who plan to implement a mix-and-match strategy for COVID-19 vaccination. Certain brands and types of vaccines may induce different side effects depending on the sequence of dosage for each participant. However, no major differences in concerns or perceived efficacy were experienced between same dose versus mix-and-match participants, so future healthcare implementations of such protocols are unlikely to be met with much resistance or hesitancy from the population if they are willing to get any vaccination.

Recently, Omicron has become a major strain and a cause of the worldwide 4th wave of COVID-19 [20]. Preliminary research has suggested that all currently available vaccines provide efficacy and protection against serious illness from Omicron [21]. However, the Pfizer-BioNTech and Moderna vaccines, when reinforced with a third booster, appear to be more successful at stopping infections compared to the Oxford-AstraZeneca or Janssen (Johnson & Johnson) vaccines, which do not seem to be effective at stopping the spread of Omicron [22]. Due to Omicron’s fast rate of spread across the world, the importance of a third booster shot and the consequences of mixing and matching vaccinations will be vital next steps to explore.

## Figures and Tables

**Table 1:** Demographic characteristics of participants (*N* = 1002).

Characteristics	Same Brand and Type of Vaccine (*n* = 791)	Different Brand but Same Type of Vaccine (*n* = 164)	Different Brand and Different Type of Vaccine (*n* = 47)
Age (years), mean (SD)	30.7 (10.6)	32.0 (10.4)	45.4 (11.2)
Age range (years)	18–77	18–68	18–65
**Gender**			
Female	393 (49.7%)	75 (45.7%)	22 (46.8%)
Male	382 (48.3%)	89 (54.3%)	25 (53.2%)
Other (e.g., non-binary, genderqueer)	16 (2.0%)	0 (0.0%)	0 (0.0%)
**Race**			
White or Caucasian	451 (57.0%)	110 (67.1%)	39 (83.0%)
East Asian	116 (14.7%)	19 (11.6%)	2 (4.3%)
South Asian or Indian	55 (7.0%)	11 (6.7%)	5 (10.6%)
Southeast Asian	55 (7.0%)	11 (6.7%)	0 (0.0%)
Black or African American	39 (4.9%)	2 (1.2%)	1 (2.1%)
Hispanic or Latino	26 (3.3%)	4 (2.4%)	0 (0.0%)
Mixed or Multi-racial	22 (2.8%)	4 (2.4%)	0 (0.0%)
West Asian or Middle Eastern	21 (2.7%)	2 (1.2%)	0 (0.0%)
Native Canadian or Indigenous	5 (0.6%)	1 (0.6%)	0 (0.0%)
Native Hawaiian or other Pacific Islander	1 (0.1%)	0 (0.0%)	0 (0.0%)
**Marital Status**			
Single	505 (63.8%)	96 (58.5%)	9 (19.1%)
Married	268 (33.9%)	62 (37.8%)	36 (76.6%)
Divorced, separated, widowed	18 (2.3%)	6 (3.7%)	2 (4.3%)
**Have children**			
Yes	202 (25.5%)	43 (26.2%)	24 (51.1%)
No	589 (74.5%)	121 (73.8%)	23 (48.9%)
**Practice some type of religion**			
Yes	307 (38.8%)	64 (39.0%)	26 (55.3%)
No	484 (61.2%)	100 (61.0%)	21 (44.7%)
**Household income**			
$0 to $29,999 CAD	101 (12.8%)	22 (13.4%)	5 (10.6%)
$30,000 to $49,999 CAD	93 (11.8%)	21 (12.8%)	3 (6.4%)
$50,000 to $74,999 CAD	184 (23.3%)	44 (26.8%)	6 (12.8%)
$75,000 to $99,999 CAD	133 (16.8%)	32 (19.5%)	7 (14.9%)
$100,000 to $149,999 CAD	174 (22.0%)	30 (18.3%)	16 (34.0%)
$150,000 to $199,999 CAD	58 (7.3%)	9 (5.5%)	5 (10.6%)
$200,000 CAD and over	48 (6.1%)	6 (3.7%)	5 (10.6%)
**Annual influenza vaccine frequency**			
Every year	187 (23.6%)	40 (24.4%)	15 (31.9%)
Almost every year	155 (19.6%)	40 (24.4%)	15 (31.9%)
Sometimes (every few years)	234 (29.6%)	35 (21.3%)	11 (23.4%)
Never	215 (27.2%)	49 (29.9%)	6 (12.8%)
**Ever been infected with COVID-19**			
Yes	35 (4.4%)	7 (4.3%)	2 (4.3%)
No	756 (95.6%)	157 (95.7%)	45 (95.7%)
**1st COVID-19 vaccine type**			
Pfizer-BioNTech (BNT162b2)	587 (74.2%)	136 (82.9%)	1 (2.1%)
Moderna (mRNA-1273)	188 (23.8%)	27 (16.5%)	3 (6.4%)
Oxford-AstraZeneca (ChAdOx1)	16 (2.0%)	1 (0.6%)	43 (91.5%)
Janssen (Johnson & Johnson) (Ad26.COV2.S)	0 (0.0%)	0 (0.0%)	0 (0.0%)
2nd COVID-19 vaccine type			
Pfizer-BioNTech (BNT162b2)	587 (74.2%)	27 (16.5%)	19 (40.4%)
Moderna (mRNA-1273)	188 (23.8%)	136 (82.9%)	24 (51.1%)
Oxford-AstraZeneca (ChAdOx1)	16 (2.0%)	0 (0.0%)	3 (6.4%)
Janssen (Johnson & Johnson) (Ad26.COV2.S)	0 (0.0%)	1 (0.6%)	1 (2.1%)

**Table 2:** Self-reported side effects as a function of severity level after 1st vaccine (*N* = 1002).

Side Effect (Mean from 0 = “*Did Not Experience*”, to 3 = “*Severe and Hospitalized*”)	Same Brand and Type of Vaccine (*n* = 791)	Different Brand but Same Type of Vaccine (*n* = 164)	Different Brand and Different Type of Vaccine (*n* = 47)
Pain, redness, or swelling on the arm where you got the needle	0.82	0.80	0.77
Tiredness or fatigue	0.67	0.57	0.94
Muscle or body pain (e.g., joint pain, muscle aches, or soreness)	0.58	0.53	0.85
Headache	0.32	0.26	0.77
Chills	0.17	0.16	0.64
Fever	0.17	0.19	0.47
Nausea	0.09	0.06	0.28
Abdominal pain	0.02	0.01	0.21
Sore throat	0.04	0.03	0.13
Chest pain or pressure	0.04	0.01	0.11
Shortness of breath	0.03	0.02	0.09
Cough	0.03	0.02	0.06
Loss of taste or smell	0.01	0.01	0.09

Note. Cell numbers represent the average amount of reported side effects multiplied by the average severity level (e.g., 0, 1, 2, 3).

**Table 3:** Self-reported side effects as a function of severity level after 2nd vaccine (*N* = 1002).

Side Effect (Mean from 0 = “*Did Not Experience*”, to 3 = “*Severe and Hospitalized*”)	Same Brand and Type of Vaccine (*n* = 791)	Different Brand but Same Type of Vaccine (*n* = 164)	Different Brand and Different Type of Vaccine (*n* = 47)
Pain, redness, or swelling on the arm where you got the shot	0.80	0.86	0.70
Tiredness or fatigue	0.82	0.99	0.81
Muscle or body pain (e.g., joint pain, muscle aches, or soreness)	0.70	0.87	0.64
Headache	0.45	0.72	0.55
Chills	0.34	0.62	0.30
Fever	0.34	0.57	0.34
Nausea	0.16	0.25	0.15
Abdominal pain	0.03	0.04	0.02
Sore throat	0.08	0.08	0.11
Chest pain or pressure	0.04	0.04	0.04
Shortness of breath	0.04	0.03	0.04
Cough	0.06	0.08	0.04
Loss of taste or smell	0.01	0.02	0.00

Note. Cell numbers represent the average amount of reported side effects multiplied by the average severity level (e.g., 0, 1, 2, 3).

**Table 4:** Total average amount of self-reported side effects after each vaccination by brand of vaccine (*N* = 1002).

Brand	Same Brand and Type of Vaccine (*n* = 791)	Different Brand but Same Type of Vaccine (*n* = 164)	Different Brand and Different Type of Vaccine (*n* = 47)
1st COVID-19 vaccine type			
Pfizer-BioNTech (BNT162b2)	2.43	2.49	--
Moderna (mRNA-1273)	3.17	2.44	--
Oxford-AstraZeneca (ChAdOx1)	2.94	--	4.09
Janssen (Johnson & Johnson) (Ad26.COV2.S)	--	--	--
2nd COVID-19 vaccine type			
Pfizer-BioNTech (BNT162b2)	2.70	2.70	2.84
Moderna (mRNA-1273)	4.58	4.33	3.88
Oxford-AstraZeneca (ChAdOx1)	2.31	--	--
Janssen (Johnson & Johnson) (Ad26.COV2.S)	--	--	--

Note. Table cells with a “--” symbol represent ≤ 3 participants, for which it would be misinformative to list their averages.

## Data Availability

The data presented in this study are available on request from the corresponding author.
